# Biological invasions as a selective filter driving behavioral divergence

**DOI:** 10.1038/s41467-022-33755-2

**Published:** 2022-10-11

**Authors:** David G. Chapple, Annalise C. Naimo, Jack A. Brand, Marcus Michelangeli, Jake M. Martin, Celine T. Goulet, Dianne H. Brunton, Andrew Sih, Bob B. M. Wong

**Affiliations:** 1 School of Biological Sciences, Monash University, Clayton, VIC 3800 Australia; 2 Department of Environmental Science and Policy, University of California at Davis, Davis, CA USA; 3 Department of Wildlife, Fish and Environmental Studies, Swedish University of Agricultural Sciences, Umeå, Sweden; 4 School of Natural Sciences, Massey University, Auckland, New Zealand

**Keywords:** Animal behaviour, Invasive species, Behavioural ecology

## Abstract

Biological invasions are a multi-stage process (i.e., transport, introduction, establishment, spread), with each stage potentially acting as a selective filter on traits associated with invasion success. Behavior (e.g., exploration, activity, boldness) plays a key role in facilitating species introductions, but whether invasion acts as a selective filter on such traits is not well known. Here we capitalize on the well-characterized introduction of an invasive lizard (*Lampropholis delicata*) across three independent lineages throughout the Pacific, and show that invasion shifted behavioral trait means and reduced among-individual variation—two key predictions of the selective filter hypothesis. Moreover, lizards from all three invasive ranges were also more behaviorally plastic (i.e., greater within-individual variation) than their native range counterparts. We provide support for the importance of selective filtering of behavioral traits in a widespread invasion. Given that invasive species are a leading driver of global biodiversity loss, understanding how invasion selects for specific behaviors is critical for improving predictions of the effects of alien species on invaded communities.

## Abstract (web summary)

Invasive species are a leading driver of global biodiversity loss. Here, the authors show that the process of invasion itself can promote behavioral changes important to the success of widespread invaders, with implications for understanding the effects of alien species on invaded communities.

## Introduction

Humans are key drivers of global environmental change^1,2^. Anthropogenic activities have redistributed the world’s biota and mediated species colonization of regions beyond their native range^3,4^. The consequences of these biological introductions are severe. Invasive species can disrupt ecological communities^5^, drive population declines and species extinctions^6,7^, and continue to cost the global economy billions of dollars every year^8,9^. Yet, despite the great number of cases and the severity of their impacts, only a small fraction of species that undergo human-assisted transportation will establish and become invasive^10,11^. Thus, identifying the traits that are selectively favored in invasive populations, and how they mediate invasion success, is of significant environmental and economic concern^12^.

Successful invasion is a multi-stage process, and each stage (i.e., transport, introduction, establishment, spread) represents a new challenging circumstance that species go through, and in which they can succeed or fail^13^. In this regard, an exciting, but untested, idea is that some introduced species may already be primed to succeed as the invasion process itself could act as a sequential selective filter promoting biological traits associated with invasion success^14,15^. It is well established that behavior can play a key role in mediating species invasions^14,16^, but whether invasion acts as a selective filter on behavioral traits is not well known^17,18^. Previous research has shown that behavioral traits facilitating invasive range expansions can be positively selected during invasions and mechanisms such as spatial sorting and subsequent interbreeding of highly dispersive individuals at the range-edge (i.e., Olympic Village effect) can further promote this process^19–22^. Moreover, the exposure to, or release from, new selective pressures (e.g., novel competitors, predators, or parasites) may also contribute to shape invasive species behavioral shifts in the latter stages of the invasion process (i.e., enemy release hypothesis)^23,24^. However, unlike post-establishment spatial sorting, selective filtering of behavioral traits may also occur during the initial stages of invasion (i.e., transport, establishment), resulting in substantial effects on the behavior of founder populations that persist long after establishment, even in the absence of any dispersal within the invasive range^14^. The impacts of selective filtering may be further exacerbated where there is a significant separation between the native and invasive ranges, leading to minimal gene flow between the two populations^25^. Thus, both initial selective filtering and post-establishment responses to invasive range environmental pressures represent distinct processes playing an important role in the evolution of behavioral phenotypes in invasive populations. Yet, despite the likely role of selective filtering in driving behavioral divergence during invasions, to our knowledge convincing evidence of a selective filter acting on behavioral traits during species invasions has not been shown^17,18^.

Here, we capitalized on a well-characterized biological invader, the delicate skink (also known as the plague skink or rainbow skink; *Lampropholis delicata*), to investigate the role of selective filtering in driving behavioral variation across the species global invasive range. The delicate skink is a small lizard species native to eastern Australia^26^ that has successfully invaded three regions across the Pacific. Importantly, our previous molecular work provides a robust reconstructed invasion history for the species, making it an ideal candidate for investigating selection on behavior during the invasion process^27^. On the Hawaiian Islands, delicate skinks invaded via human-mediated dispersal from the Brisbane region in approximately 1905^27^. The species was restricted to Oahu until shortly after WWII, when over an ~12-year period (1963–1975), it was detected on the other five main Hawaiian islands^27^. In New Zealand, delicate skinks colonized in the early 1960s via a shipment of railway sleepers from the Tenterfield region^27,28^. It was restricted to the Auckland region for ~15 years until it spread rapidly across the North Island, predominantly via human-mediated dispersal followed by secondary natural range expansion (Hamilton: 1978, Whangarei: 2002, Edgecumbe: 2007)^27,29^. The species is still actively expanding its range across both the North and South Islands of New Zealand^30^. The most recent invasion of the delicate skink was to Lord Howe Island, where it colonized in the 1980s as a stowaway in cargo and supplies from the Coffs Harbour region^27,31,32^. Its subsequent spread across the island was driven by additional introductions, and subsequent genetic admixture, from other native range source regions (Brisbane, Sydney, Tenterfield)^27,31,32^.

The delicate skink is highly adept at human-mediated dispersal. It is the only Australian lizard species that has become invasive overseas, and it has done so on multiple occasions, with subsequent human-assisted spread within each invasive region^27,29^. For instance, New Zealand biosecurity records indicate that it is one of the most frequent lizard species intercepted arriving as a stowaway from Australia^28^ and is predominantly spread via human-mediated dispersal within New Zealand^29^. We have previously hypothesized that behavior may drive the proficiency of the delicate skink as a stowaway, as it is more exploratory than non-invasive congenerics^33,34^.

**Fig. 1:**
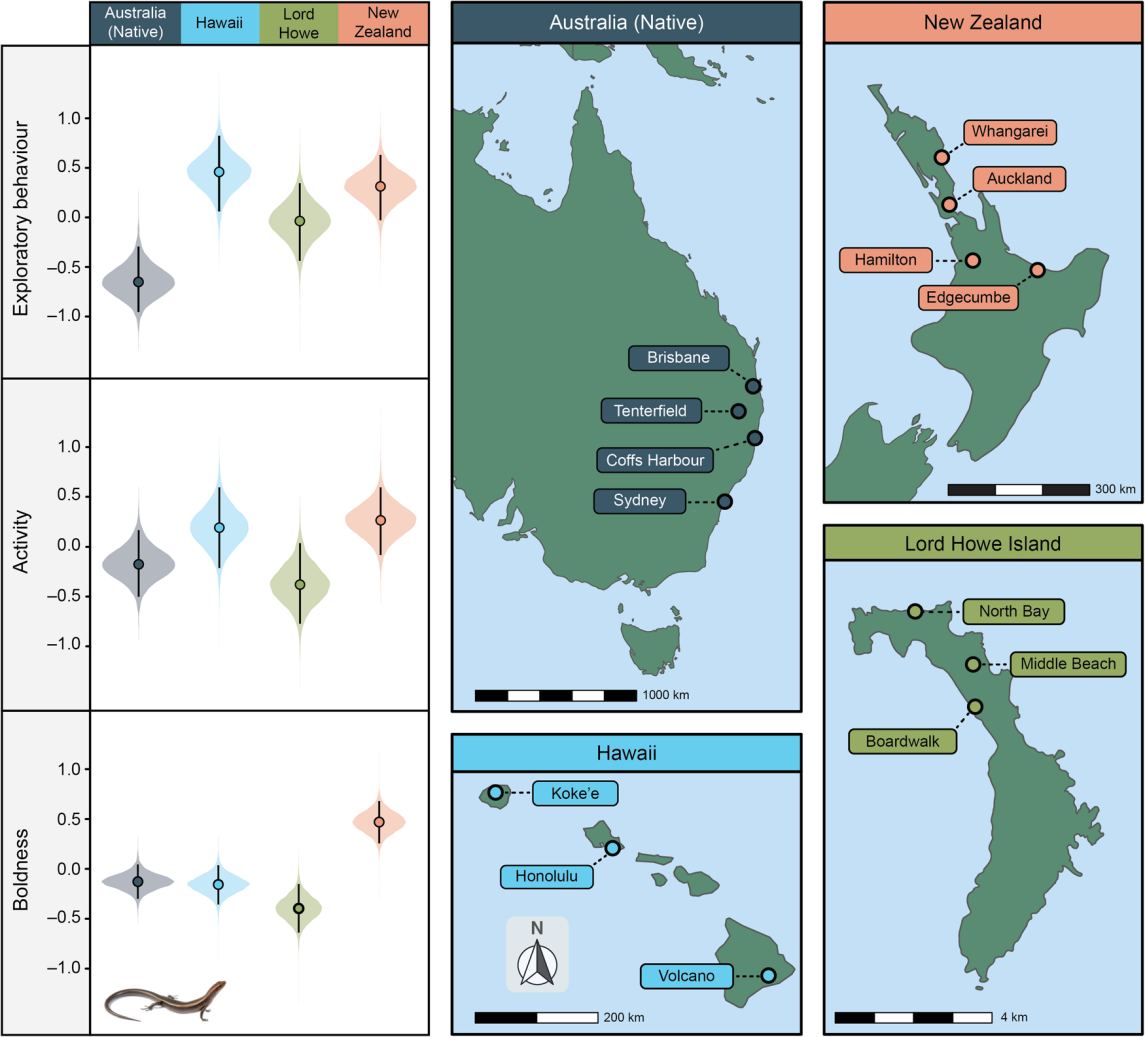
Schematic diagrams of both native (Australia) and invasive (Hawaii, Lord Howe Island, and New Zealand) delicate skink populations collected in this study. Result plots show average regional differences in exploratory behavior (i.e., time spent exploring the barrier), activity (i.e., the number of transitions between grid squares), and boldness (i.e., re-emergence latency; axis inverted) between Australia (grey; *n* = 167 skinks), Hawaii (blue; *n* = 118), Lord Howe Island (green; *n* = 92), and New Zealand (pink; *n* = 143). Boldness scores were inverted so that higher values represent bolder lizards. All behavioral scores are presented in standardized units. For each results graph, filled circles represent posterior medians, vertical error bars denote 95% credible intervals, and plot width represents probability density. Source data are provided as a Source Data file.

To test the selective filter hypothesis, we capitalized on the invasion of the delicate skink across the Pacific. Previous research has found that invasive species are often more exploratory, active, and bolder than their native range counterparts^18,35–38^. Here, it is suggested that these traits may make species more likely to find their way onto transport vectors in the first place, and/or more likely to disperse to locate suitable habitat and resources once introduced into their new range^14^. Therefore, it is expected that invasions may act as a ‘selective filter’ on these traits, resulting in invasive populations being more exploratory, active, and bolder than native range conspecifics. However, prior studies have mainly investigated behavioral changes in a single invasive population, making it difficult to disentangle selective filtering during invasion from initial founder effects. We, therefore, systematically and repeatedly measured exploratory behavior, activity, and boldness in 520 lizards from 14 populations, across three independent invasive lineages (Hawaii, New Zealand, Lord Howe Island), as well as their Australian native range source regions (Sydney, Coffs Harbour, Tenterfield, and Brisbane; Fig. 1; Supplementary Table 1). Within this framework, while the occurrence of similar founder effects resulting from multiple independent invasions is unlikely, consistency in patterns across independent invasive lineages is expected under the selective filtering hypothesis. Thus, by investigating the occurrence of a shared behavioral pattern across the distinct skink lineages within the three invasive ranges, we aim to shed light on the key signatures of selection during the invasion process.

We first used a broad, regional-level analysis to determine how behavior has changed after introduction into Hawaii, New Zealand, and Lord Howe Island. We predicted that invasion would have driven a shift in behavioral trait means, with introduced populations being more exploratory, active, and bolder than lizards from the native range. Further, if invasion acts as a selective filter on these traits where only the most exploratory, active, and boldest individuals are introduced, we expected that lizards in invasive populations would be more behaviorally similar to each other than their native range counterparts. That is, we predicted that there would be a reduction in among-individual variation in all three invasive lineages when compared to native range populations. In addition to the predictions of the selective filter hypothesis, previous research has suggested that behavioral plasticity may be one way in which animals cope with environmental change^39^. To this end, we expected that there would be an increase in behavioral plasticity in invasive populations. In line with previous research, we quantified behavioral plasticity as within-individual behavioral variation (i.e., a form of reversible behavioral plasticity) that can be due to either behavioral changes in response to variation in environmental conditions, or within-individual behavioral variation even in the same conditions (i.e., low behavioral rigidity)^40–42^. The former can be a valuable immediate response to novel conditions, and the latter can allow further adjustments to repeated exposures to the same novel conditions. Both should be valuable for individuals invading new habitats. Here, we tested for whether behavioral plasticity differs between native and invasive populations. In conjunction with the broad-scale comparisons across the Pacific (i.e., regional-level analysis), the predictions of the selective filter hypothesis are also examined at a finer spatial scale by exploiting the well-characterized range expansion of invasive delicate skinks within New Zealand (i.e., within-region analysis). We expected that the most recently established New Zealand populations would show an increase in the means of the focal behavioral traits, a reduction in among-individual variation, and an increase in behavioral plasticity relative to longer-established conspecifics.

## Results and discussion

The results of the present study are in agreement with the predictions of the selective filter hypothesis, suggesting that this mechanism might have played an important role in driving the behavioral shifts observed in the invasive populations of the delicate skink (Fig. 1; Supplementary Tables 6–8). In the broad, regional-level analysis, we found a substantial increase in exploratory behavior after the introduction of skinks into all three invasive regions (i.e., Hawaii, New Zealand, Lord Howe Island), relative to native Australian populations (Fig. 1; Supplementary Table 6). Similarly, invasive lizards from New Zealand were substantially bolder than their native range counterparts, and were slightly more active (even if credible intervals marginally overlapped zero) than source Australian populations (Fig. 1; Supplementary Tables 7 and 8). However, this was not the case for invasive skinks from Hawaii, which showed no substantial difference in either their activity or boldness when compared to native Australian populations (Fig. 1; Supplementary Tables 7 and 8). Moreover, while Lord Howe Island skinks were slightly less bold than native range populations (credible intervals marginally overlapped zero), there was little difference between the two regions in their activity levels (Fig. 1; Supplementary Tables 7 and 8). Interestingly, we found that skinks from all three invasive regions lacked the well-characterized activity-exploration behavioral syndrome previously found in native Australian populations^43^. More specifically, while Australian populations demonstrated an activity–exploration behavioral syndrome (r [95 % CI] = 0.414 [0.215, 0.607]), this was not found in skinks from invasive Hawaiian (0.275 [–0.091, 0.642]), New Zealand (0.250 [–0.177, 0.644]), or Lord Howe Island (0.078 [–0.400, 0.542]) ranges, suggesting that invasion has disrupted this behavioral syndrome. There was no evidence of correlations between any other behavioral traits from any of the native or invasive regions.

**Fig. 2:**
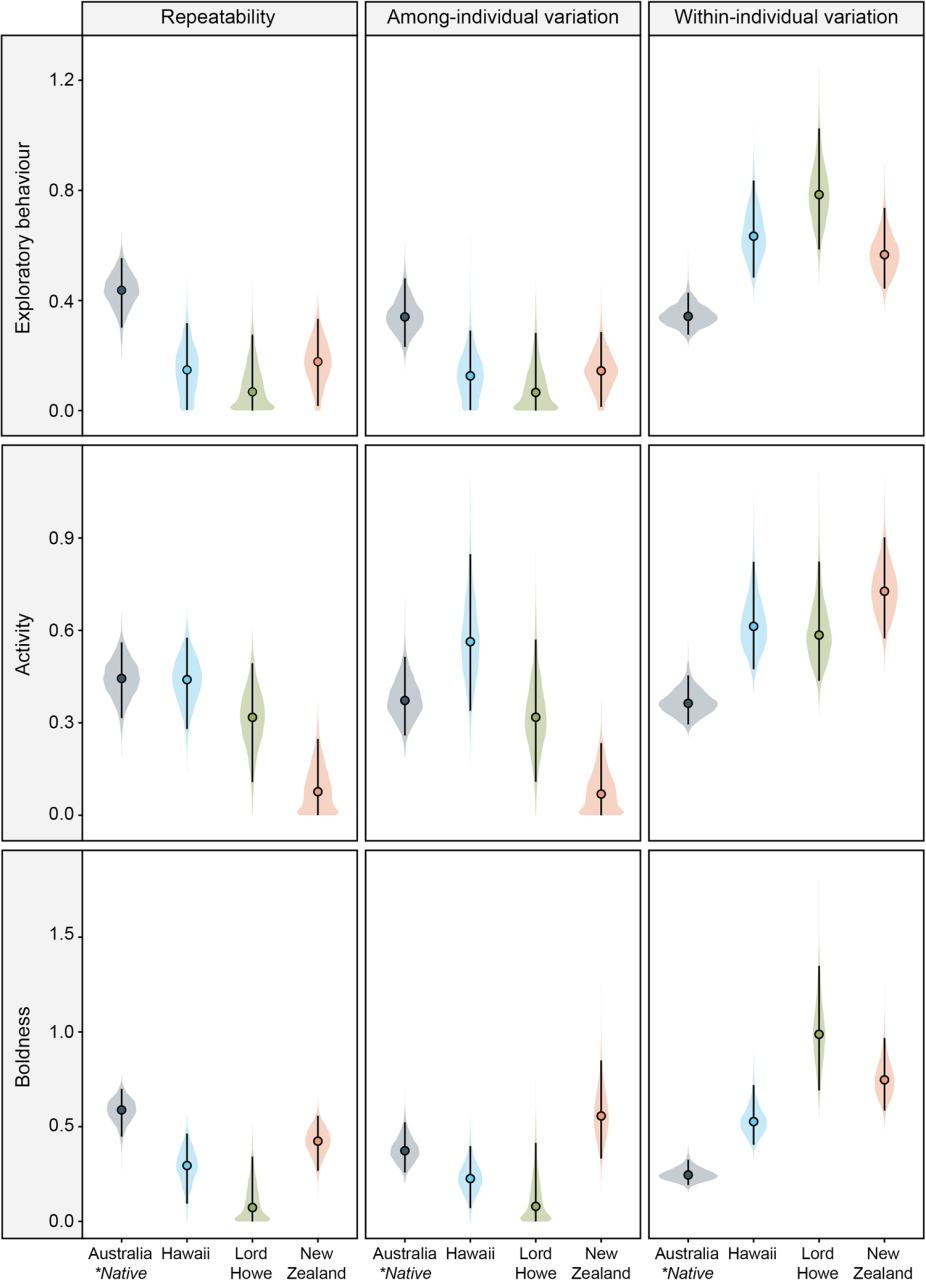
Adjusted, short-term repeatability and variance estimates (among- and within-individual) for exploratory behavior (i.e., time spent exploring the barrier), activity (i.e., number of transitions between grid squares), and boldness (i.e., re-emergence latency) of lizards from native Australian populations (grey; *n* = 167 skinks), as well as skinks from their invasive range in Hawaii (blue; *n* = 118), Lord Howe Island (green; *n* = 92), and New Zealand (pink; *n* = 143). For each graph, filled circles represent the median variance/repeatability estimates extracted from linear mixed-effects models, vertical error bars denote 95% credible intervals, and plot width represents probability density. Source data are provided as a Source Data file.

Using variance partitioning, we then investigated whether invasion resulted in lower among-individual behavioral variance within invasive populations—a key prediction of the selective filter hypothesis (Table 1; Supplementary Tables 9–11). We found that skinks from all three independent invasive regions (i.e., Hawaii, New Zealand, Lord Howe Island) exhibited a substantial decrease in among-individual variation in their exploratory behavior relative to native Australian populations (Table 1; Fig. 2). Similarly, invasive New Zealand skinks demonstrated lower among-individual variation in their activity than their native range counterparts (Table 1; Fig. 2). However, there were no clear differences in among-individual variation in activity between native Australian lizards and introduced populations from either Hawaii or Lord Howe Island (Table 1; Fig. 2). Further, there were no apparent differences in among-individual variation in boldness between Australian populations and skinks from any of the three invasive regions (i.e., Hawaii, New Zealand, Lord Howe Island; Table 1; Fig. 2).

**Table 1:** The effect size (±95% CI) of the magnitude difference in among-individual variation (ΔV_A_), within-individual variation (ΔV_W_), and repeatability (ΔR) of exploration, activity, and boldness of lizards from Australia (AUS; *n* = 167 skinks), Hawaii (HAW; *n* = 118), Lord Howe Island (LHI; *n* = 92), and New Zealand (NZ; *n* = 143)

	Exploration			Activity			Boldness		
Contrast	ΔV_A_	ΔV_W_	ΔR	ΔV_A_	ΔV_W_	ΔR	ΔV_A_	ΔV_W_	ΔR
AUS – HAW	**0.21**	**−0.30**	**0.29**	−0.19	**−0.26**	0.01	0.15	**−0.29**	**0.29**
	**(0.02, 0.40)**	**(−0.49, −0.11)**	**(0.09, 0.49)**	(−0.49, 0.09)	**(−0.46, −0.07)**	(−0.17, 0.19)	(−0.05, 0.36)	**(−0.46, −0.13)**	**(0.08, 0.52)**
AUS – LHI	**0.26**	**−0.44**	**0.35**	0.06	**−0.23**	0.13	0.26	**−0.75**	**0.48**
	**(0.05, 0.45)**	**(−0.67, −0.21)**	**(0.16, 0.54)**	(−0.20, 0.32)	**(−0.45, −0.03)**	(−0.08, 0.35)	(−0.03, 0.49)	**(−1.07, −0.41)**	**(0.24, 0.68)**
AUS – NZ	**0.20**	**−0.23**	**0.26**	**0.30**	**−0.36**	**0.36**	−0.19	**−0.50**	0.16
	**(0.02, 0.39)**	**(−0.39, −0.06)**	**(0.07, 0.45)**	**(0.12, 0.49)**	**(−0.55, −0.18)**	**(0.17, 0.53)**	(−0.47, 0.10)	**(−0.72, −0.32)**	(−0.02, 0.35)

Contrasts in bold are those with 95% CI’s that did not include zero.

Taken together, these results suggest that invasion may favor more risk-prone (i.e., exploratory, active, and bold) behavioral types by promoting shifts in behavioral trait means, and changes in behavioral variation. This was especially true for exploratory behavior, where skinks from all three independent invasive ranges (i.e., Hawaii, New Zealand, and Lord Howe Island) were more exploratory and demonstrated substantial reductions in among-individual variation compared to native range conspecifics. We contend that these findings are consistent with the selective filter hypothesis and not merely due to founder effects during the initial introduction. Specifically, under a founder effects framework, we would have expected the directionality of trait differences between native and invasive populations to be random. Instead, we found consistent patterns across all three independent invasive lineages (at least for exploratory behavior). Similarly, the decrease in among-individual variation in exploratory behavior was not found for all behavioral traits in all invasive populations, again suggesting that reduced among-individual variance may not necessarily be due to founder effects during the initial invasion. Moreover, the consistent patterns in exploratory behavior were even found in the Lord Howe Island invasive populations, where repeated introductions from multiple sources, and subsequent genetic admixture are expected to have reduced any local adaptation and/or runaway selection for invasion promoting behavioral traits (i.e., Olympic Village effect)^19,20^. Collectively, these results suggest that invasion itself may act as a selective filter promoting risk-prone behavioral types.

However, at which stage of the invasion this selection operates at is unknown. For example, this selective filter may occur during the uptake and transportation stage, where only the most exploratory individuals find their way onto transport vectors (i.e., shipping, air, etc.) in the first place^14^. Indeed, previous research has suggested that the increased exploratory tendencies of delicate skinks may make them more likely to become ensnared in transport vectors and moved to regions beyond their native range^33^. These findings, together with biosecurity records showing that most lizards are transported as single individuals^28^, suggests that pre-establishment selection for increased exploratory behavior may occur during the initial stages of invasion. Whether such traits are still adaptive long after establishment when populations eventually become subject to predator-induced and density-dependent natural selection is not clear^44,45^. Future research measuring the behavior of lizards intercepted during initial transit and after establishment will be needed to investigate at which stage the selective filtering of behavioral types may occur, and how selection may act on these behavioral types once populations become well-established.

**Fig. 3:**
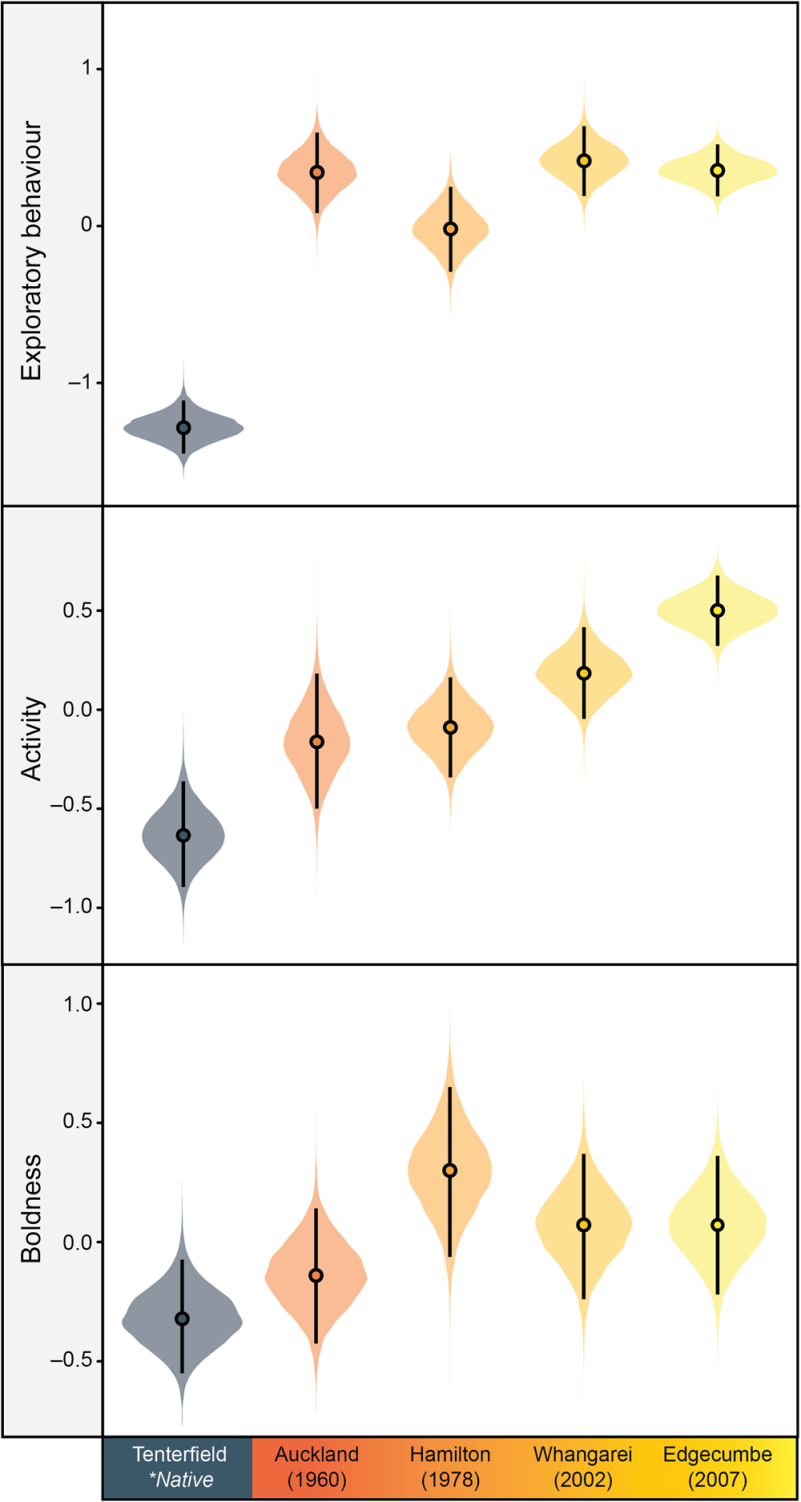
Differences in average-level exploratory behavior (i.e., time spent exploring the barrier), activity (i.e., number of transitions between grid squares), and boldness (i.e., re-emergence latency; axis inverted) between the native Tenterfield source population (grey; *n* = 30 skinks), and invasive New Zealand skinks from Auckland (red; *n* = 31), Hamilton (orange; *n* = 43), Whangarei (light-orange; *n* = 33), and Edgecumbe (yellow; *n* = 36). Boldness scores were inverted so that higher values represent bolder lizards. All behavioral scores are presented in standardized units. For each graph, filled circles represent posterior medians, vertical error bars denote 95% credible intervals, and plot width represents probability density. Source data are provided as a Source Data file.

We found similar results from the fine-scale, in-depth analysis of population differences within the invasive New Zealand range (Supplementary Tables 15–17). Specifically, all invasive New Zealand populations were more active than their native range source, with the most recently established populations (e.g. Whangarei, Edgecumbe) towards the edge of their range showing the greatest difference (Fig. 3; Supplementary Tables 16). These results suggest that the sequential selective filtering of populations during human-mediated dispersal within the invasive New Zealand range^29^ may select for increasingly active skinks in the most recently established populations (but see subsequent discussion on the potential role of spatial sorting). Furthermore, most invasive New Zealand populations were bolder and more exploratory than their native range source (Fig. 3; Supplementary Tables 15 and 17). However, unlike for activity, there was no clear evidence that this increased in the more recently established populations. Additionally, there was a general trend towards lower among-individual variation in activity in more recently established populations (Supplementary Fig. 1; Supplementary Table 19). In contrast, we found no clear differences when comparing each invasive New Zealand population to the Australian source in among-individual variation in both exploratory behavior and boldness (Supplementary Fig. 1; Supplementary Tables 18 and 20).

Although our findings are concordant with predictions of the selective filter hypothesis, it is important to note that post-establishment processes may also contribute to an increase in risk-prone behavioral phenotypes in invasive populations. For instance, post-establishment spread dynamics could also facilitate behavioral change in invasive populations. For example, previous research investigating the spread of introduced cane toads (*Rhinella marina*) across northern Australia found that spatial sorting of phenotypes facilitates the evolution of increasingly exploratory, active, and bold individuals at the range edge of the invasion^21,46,47^. Indeed, spatial sorting and subsequent interbreeding of risk-prone individuals (i.e., Olympic Village effect) may also be responsible for the current results, whereby the most recently established New Zealand populations towards the edge of their range were the most active.

However, we think it unlikely that spatial sorting is largely responsible for the observed shift in the behavior of delicate skinks across their invasive range. Firstly, within the New Zealand invasion, the increase in risk-prone behavioral types (at least for exploratory behavior and activity) was seen even in the initial founding Auckland population (established ~ 1960’s), suggesting a potential role for pre-establishment selective filtering in the observed behavioral shift. Secondly, our previous research has shown that invasive delicate skinks in New Zealand often spread via human-mediated jump dispersal between disjunct locations, rather than solely via natural range expansion^27,29^. This is similar to Hawaii, where delicate skinks have spread between islands via human-mediated dispersal^27^. Finally, delicate skinks now have a nearly continuous distribution across Lord Howe Island^27^. However, despite the island’s small size (~11 km long, ~2 km wide; Fig. 1), there is a clear spatial structuring of haplotypes from different native source regions across the island^27^. This suggests that the colonization of Lord Howe Island was driven by multiple, separate introductions from various Australian source populations, rather than via natural range expansion from an initial founder population^27^. Together, these results highlight that spatial sorting during natural range expansion is unlikely to be largely responsible for the observed behavioral shifts in invasive delicate skinks.

Similarly, reduced predation pressure and/or competition in the invasive range may also promote risk-prone behavioral phenotypes (i.e., enemy release hypothesis)^23^. Indeed, enemy release within the invasive range may reduce the fitness costs of risky behavioral strategies, resulting in introduced populations being more risk-prone than their native counterparts. While we cannot rule out the possibility that enemy release may have facilitated behavioral shifts in the invasive populations, we again believe that this process is unlikely to be solely responsible for the current pattern of results. For example, Hawaii, Lord Howe Island, and New Zealand all have abundant avifauna that likely predate upon invasive delicate skinks. Indeed, there are substantial populations of invasive Australian magpies (*Gymnorhina tibicen*) in New Zealand^48^, which are known to predate on small skinks in their native range^49^. Further, during fieldwork on Lord Howe Island, we often observed invasive delicate skinks being predated upon by native Lord Howe Island currawongs (*Strepera graculina crissalis*; A.C. Naimo, *pers. obs*.), suggesting that delicate skinks are a likely target for predation in their invasive range. Thus, while both spatial sorting and enemy release likely play a part in driving the current pattern of results (and are potentially responsible for observed behavioral differences between the separate invasive regions where the intensity of these processes may differ), we contend that it is unlikely that these processes are predominantly responsible for the consistent behavioral shifts found across multiple, independent invasive lineages of the delicate skink. Together, the present results suggest that pre-establishment selective filtering for risk-prone behavioral types is a likely mechanism in driving the behavioral divergence of invasive delicate skinks. Understanding the probable interplay between both pre- and post-establishment processes in facilitating behavioral change in introduced populations will be an important topic for future research.

We also found evidence for increased within-individual variation (i.e., behavioral plasticity) in the introduced populations (Fig. 2; Table 1; Supplementary Tables 9–11). Indeed, lizards from all three invasive ranges (i.e., Hawaii, New Zealand, and Lord Howe Island) were more behaviorally plastic in their exploratory behavior, activity, and boldness than native Australian populations (Table 1; Fig. 2). This increased within-individual behavioral variation in the introduced populations resulted in a decrease in behavioral repeatability in the invasive range compared to native populations—a finding that was generally consistent across all three invasion pathways (Table 1; Fig. 2). This pattern was also found when investigating fine-scale population differences in behavioral plasticity within the New Zealand range (Supplementary Tables 18–21). Indeed, even if differences between New Zealand populations were less marked and there was substantial uncertainty around the estimates, we found that New Zealand populations were generally more behaviorally plastic in their boldness and exploratory behavior than their native range source region, and this difference (at least for exploratory behavior) seemed to be greatest in more recently established populations (Supplementary Tables 18 and 20–21; Supplementary Fig. 1). Similarly, there was also a trend towards increased within-individual variation in activity in invasive New Zealand populations compared to their native source (Supplementary Tables 19 and 21). Again, the observed increase in within-individual variance of skinks from New Zealand was accompanied by a general reduction in the repeatability of exploratory behavior, boldness, and activity compared to their native Australian source population (Supplementary Tables 18–21; Supplementary Fig. 1).

These findings clearly demonstrate that invasion promotes increased within-individual behavioral variation (i.e., behavioral plasticity). While the magnitude of the effect varied amongst populations, the pattern was true for all behavioral traits in all three independent invasion lineages. This suggests that increased behavioral plasticity may be one way in which organisms cope with changing environmental conditions during biological invasions^39,50^, resulting in invasive populations being, in general, more phenotypically plastic than their native range counterparts. Indeed, behavioral plasticity may buffer individuals against novel selection pressures experienced within the invasive range by allowing them to rapidly adjust their behavior to current environmental conditions^51^. This flexibility in behavior may promote population stability and persistence, particularly during the early stages of invasion, where invaders are characterized by small population sizes that are susceptible to environmental and demographic stochasticity^51,52^. Whether the increased behavioral plasticity seen in invasive populations is an evolved adaptive response or is due to evolutionarily novel conditions experienced by invaders during development is not clear and will require further research.

Collectively, these results emphasize the importance of behavior in invasion biology and suggest that biological invasions may favor increasingly risk-prone individuals that are particularly adept at altering their behavior in response to environmental change. This may pose a particular threat to invaded communities. Indeed, previous research has found associations between risk-prone behavioral types and competitive ability^53,54^, with potential implications for predator-prey interactions^55,56^, as well as community structure and trophic cascades^57^. Thus, selection for increasingly exploratory, active, and bold invaders may have an outsized effect on local native species. Given obvious consequences for invasion dynamics, our findings also underscore the importance of monitoring sensitive trade routes for potential stowaways if more risk-taking individuals have a higher propensity to become accidentally transported beyond their native range. More generally, as invasive species are a leading driver of global biodiversity loss^6,58^, understanding how invasion selects for specific behavioral phenotypes in invading populations may allow us to better predict the effects of alien species on invaded communities.

## Methods

The research was conducted in accordance with all relevant ethical approvals (University of California, Davis Animal Ethics Committee Protocol No. 211194, Monash University School of Biological Sciences Animal Ethics Committee Protocol No. 16736, Massey University Animal Ethics Committee Protocol No. MUAEC17/76) and collection permits (Hawaii: K2019-4044cc and EX-19-18, Lord Howe Island: LHIB 09/18, Australia: SL102160 [NSW] and 10008946 [VIC]).

### Study sites and animal husbandry

We collected 520 delicate skinks (*Lampropholis delicata*) from 14 populations across the species’ native (mainland Australia) and invasive (New Zealand, Lord Howe Island and Hawaii) range between November 2015 and August 2019 (see Supplementary Table 1). More specifically, we collected skinks from four sites across their native Australian range (*n* **=** 167; range = 27–81 lizards from each site), four sites across their invasive range in New Zealand (*n* **=** 143; range = 31–43 lizards from each site), and three sites each within the skinks’ introduced range on both Lord Howe Island (*n* = 92; range = 26–36 lizards from each site) and Hawaii (*n* = 118; range = 39–42 lizards from each site). Lizards were sourced from populations in both urban and non-urban sites within each native and invasive region. However, our previous research has found no general effect of urbanization on the exploratory behavior, activity, and boldness of delicate skinks^59^. Indeed, consistent patterns of behavior were found in the current study across multiple independent invasive lineages, all with varying degrees of urbanization, suggesting that urbanization likely played a minor role, if any, in explaining skink behavioral responses. All skinks were caught using hand capture, mealworm fishing, and passive trapping. These trapping methods have previously shown to not retain any sampling bias towards exploratory, active, or bold skinks^60^. Lizards were measured for snout-vent length (SVL) using digital calipers and marked with unique permanent identification codes using Visual Implant Elastomer (Northwest Marine Technology, Shaw Island, WA, U.S.A.). We collected adult, male skinks with complete tails (i.e., SVL > tail length) to avoid the well-documented effects of tail loss^34,61^ and gravidity^62^ on *Lampropholis* behavior. Skinks were transported in small groups within temporary housing containers via a combination of car and air travel back to animal facilities at either the Center for Aquatic Biology and Aquaculture, University of California, Davis (Hawaiian populations), Massey University, Auckland (New Zealand populations), or Monash University, Melbourne (Australian and Lord Howe Island populations). Transport times from the field site to the laboratory ranged between 1–14 days within each region. Although we cannot rule out the possibility that variation in transport times may have had an effect on behavior (as we did not record the transport times of individual lizards), it is important to point out that transport times were approximately equivalent between regions (i.e., no substantial differences in transport times between native and invasive skinks). During transport, skinks were housed within social groups in plastic containers (same as described directly below; ~7 skinks per container) and fed three times a week, with water available *ad libitum*.

As *Lampropholis delicata* is a social species, skinks were housed in groups of up to seven individuals in plastic containers (300 × 370 mm) within temperature-controlled rooms (13:11 h light/dark cycle, maintained at 22.5 ± 1 °C), in line with established protocols^63,64^. Skinks from each population were randomly assigned to housing containers, with approximately 7 separate housing containers used for each population (i.e., ~98 housing containers total). Each container was fitted with small plastic pots (width = 60 mm; depth = 120 mm) and newspapers for shelter. A basking area (130 mm diameter) was created by placing heat-tape under a terracotta tile at one end of each housing container. This created a thermal gradient (22–32 °C) that allowed all skinks to thermoregulate from 0800 to 1700 h. Similarly, UV lighting was provided above containers from 0800–1800 h. Skinks were fed a diet of crickets (*Acheta domesticus*) dusted in a vitamin supplement (Reptivite), three times a week, and water was made available ad libitum. Skinks were acclimated to these standardized laboratory conditions for at least 1 week before experiments, in line with previously established protocols^43,64^.

### Behavioral experiments

All skinks were tested for activity, exploration, and boldness following previously established methods for *Lampropholis* skinks^43,60^. These behavioral traits were chosen because they are repeatable^43,60^, have key ecological implications for native delicate skinks^65,66^, and are thought to play a mediating role in species’ invasions^14^. All lizards were tested during their normal diel activity times (~0800–1700 h) in a controlled temperature room maintained at 22.5 ± 1 °C. Skinks first performed: (i) an activity assay testing activity levels, (ii) a novel obstacle test testing exploratory behavior, and (iii) a boldness assay following a simulated predator attack (see Supplementary Table 2 for experimental timeline). All animals were subjected to the same fixed test order sequence to minimize potential differences among individuals due to carryover effects^67^. Further, within each day, the order that individual skinks completed the behavioral tests was randomized across all populations to avoid any confounding effects of time of day on population differences in behavior. Each assay was repeated after four–seven days to measure both among- and within-individual behavioral variation. Trials were video-recorded (JVC Everio GZ-E100) from above and scored blind to experimental conditions using an event-logging software designed for behavioral analyses (i.e., BORISv8^68^). All equipment was thoroughly washed between each trial with scentless detergent. Further, as *Lampropholis* skinks are known to modify their behaviors following large meals^62^, we ensured that lizards were not fed for 24 h prior to each behavioral assay. Finally, there were no instances of caudal autonomy (i.e., voluntary tail shedding) during the experiments.

Following the completion of all behavioral trials, lizards were either maintained in the laboratory for future experiments, or humanely killed in line with approved animal ethics protocols.

### Activity

To measure activity levels, skinks were randomly collected from their housing containers by hand and placed in an experimental arena (550 × 320 mm) marked with 20 equal grid squares (110 × 80 mm). Skinks were acclimated for 10 min in a transparent plastic container placed in the centre of the arena. This transparent container allowed skinks to become familiar with their surroundings prior to the start of behavioral trials. After acclimation, the transparent container was removed by hand and skinks were allowed to move freely in the arena for 20 min. We scored activity as the number of transitions between grid squares made by the skink. A lizard was considered to have transitioned between grid squares when their center of mass crossed the line separating two squares.

### Exploration

To measure an individual’s exploratory tendency, skinks were collected and placed by hand into an experimental arena (550 × 320 mm; see Activity section for details) containing a novel opaque barrier (width = 320 mm; height = 300 mm). The barrier was placed approximately 365 mm from one end of the arena (i.e., 2/3rds down the arena) and acted as a wall that divided the arena into a large (zone 1) and small (zone 2) zone. Two small gaps were located at either end of the barrier that enabled lizards to squeeze through and enter the second zone. Thus, skinks could only enter the second zone by exploring the novel barrier and finding the small gaps. At the beginning of the exploration trial, skinks were left to acclimate for 10 min in a transparent plastic container placed in the center of zone 1. After acclimation, skinks were allowed to move freely for 20 min. We measured exploratory tendency as the amount of time a skink spent exploring the barrier and the number of times that skinks passed the barrier. Skinks were considered to be exploring the barrier when they were actively moving while in contact with the barrier. Similarly, lizards were recorded as having crossed the barrier once their center of mass passed through the barrier gap separating the two zones. The amount of time exploring the barrier was highly correlated to the number of times the skink passed into zone 2. We, therefore, used the total time spent exploring the barrier as our measure of exploration, in line with previous studies in *Lampropholis* skinks^43^.

### Boldness

We measured boldness following a simulated predator threat. Individual skinks were collected and placed into an experimental arena (550 × 320 mm; see Activity section for description) fitted with a basking site at one end and a shelter site at the other. The basking site was a circular ceramic tile with a 4.5 cm radius, heated to ~35 °C by a 40 W bulb overhead. The shelter sites were the same typology as those used within lizard housing to ensure they were familiar to the focal skinks. The shelter and basking sites used during the experiment were cleaned prior to use to ensure that there was no effect of conspecific scent cues on lizard behavior. At the beginning of the trial, skinks were left to acclimate for 10 min in a transparent plastic container placed in the center of the arena. After the acclimation period, an observer simulated a predatory attack by prodding the lizard close to its tail with a rod until the lizard entered the shelter site. We then recorded the arena for 60 min, and boldness was measured as the latency for the skinks to emerge from the shelter site (i.e., center of mass out of the shelter).

### Statistical analysis

Analysis was conducted in R version 3.6.2^69^. We used the Bayesian package brms^70^ for generalized linear mixed models. Data from trials in which lizards did not move at all during the assays (2.8% of total activity data; 3.9% of total exploration data) were removed from the analysis, as these irregularities indicate an abnormal behavioral response. In addition, video-recordings failed in some behavioral trials. This resulted in a total of 1005 activity trials (mean = 1.93 measures per individual), 1021 exploration trials (mean = 1.96 measures per individual), and 891 boldness trials (mean = 1.71 measures per individual) from 520 skinks included in the analysis. The number of grid transitions (i.e., activity) and time spent exploring the barrier (i.e., exploration) were square-root transformed, while the time to emerge from the shelter (i.e., boldness) was log_10_ transformed to ensure that all models met the assumption of normality. Further, all response variables were standardized (i.e., mean = 0, SD = 1) to aid in model interpretation. Posterior predictive checks were performed to ensure adequate model fits. Similarly, we checked models for sufficient mixing via trace plots, with all models converging with low among-chain variability (Rhat = 1). We report posterior means with 95% credibility intervals (CI) for all estimated parameters (fixed and random effects).

#### Regional-level analysis

For each behavior (exploration, activity, and boldness), we compared the fit of four univariate generalized linear mixed-effects models with different random-effect structures to determine whether regions differed in both average-level trait expression and trait variation, in line with previously established methods^42,71–73^. More specifically, we created a set of four candidate models for each behavior that either hold or allow variances to differ between regions (see Supplementary Tables 3–5). We compared models where the variance components (among-individual variance [V_A_], within-individual variance [V_W_], and both V_A_ and V_W_) were allowed to vary between regions to a null model where the variances were restricted to be equal between regions. These models included:

1. Model 1 (null model): a null model where among-individual variance (V_A_) and within-individual variances (V_W_) were kept constant between regions.
2. Model 2 (among model): a model where only among-individual variance (V_A_) is calculated separately for each region.
3. Model 3 (within model): a model where only within-individual variances (V_W_) is calculated separately for each region.
4. Model 4 (both variance model): a model where both V_A_ and V_W_ are calculated separately for each region.

This approach allowed us to test for a statistical effect of region on both variance components that underlie total phenotypic variance^72^. All models contained the same fixed effect of region (Australia, Lord Howe, New Zealand, and Hawaii), while both individual lizard ID and population ID were included as random intercepts. We included population ID as a random-effects term in all models to account for any variation arising from population differences within regions. Our previous research has found no differences in body size between native and invasive delicate skinks^74^, nor any effect of lizard size on the activity, exploratory behavior, or boldness of delicate skinks when tested under standardized conditions^43,59,75^. Therefore, SVL was not included in the current models to reduce model complexity. Further, housing container ID explained little variance in preliminary models (likelihood ratio test comparing models with and without housing container ID included as a random effect for each behavioral trait; *P* always > 0.25), and therefore, was not included in the final analyses. Models were compared using both the widely applicable information criterion (WAIC), a generalization of AIC for model comparison within a Bayesian framework with lower WAIC values indicating a better model fit, as well as leave-one-out (LOO) cross-validation^76^. For each behavioral trait, the model which allowed both among- and within-individual variance to differ among regions (i.e., Model 4) provided the best fit to the data (see Supplementary Tables 3–5). We used this model (i.e., Model 4) to calculate and compare mean behavior, behavioral repeatability, and the associated variance components between regions. We calculated short-term, adjusted repeatability (R) for each region, which represents the amount of total phenotypic variation explained by among-individual differences after accounting for the fixed effects following Eq. 1:

1 $$R=\frac{{V}_{A}}{{V}_{A}+{V}_{W}+{V}_{{Pop}}}$$

where V_A_ represents the among-individual variance, V_W_ represents the within-individual variance, V_Pop_ represents the variance among populations within each region. A repeatability value higher than 0.5 would suggest that the majority of observed phenotypic variation would be due to differences among individuals^77^. We also report an estimate of the magnitude of difference in repeatability and variance components among regions (∆R, ∆V_A_, and ∆V_W_; see Table 1)^72^. These measurements provide an estimation of the effect size of the differences in variances between each regional contrast allowing for comparison between datasets^72^. All models were run with relatively uninformative priors on four chains for 5000 iterations (1000 warmup).

To examine whether there were behavioral syndromes (i.e., among-individual behavioral correlations) within each region, we ran a multivariate linear mixed model with all behaviors included as response variables. All models contained region as a fixed effect, while individual lizard ID and population ID were included random intercepts. We allowed the random intercept of lizard ID to vary among regions. The multivariate model was run with relatively uninformative priors on four chains for 10,000 iterations (1000 warmup).

#### Within-region analysis (New Zealand)

Following the regional-level analysis, we capitalized on the sequential spread and establishment of invasive populations across the New Zealand range to test whether selective filtering during introduction lead to a general decrease in trait variation and a stronger increase in trait means in more recently established populations. We used an almost identical statistical approach to that described above. More specifically, we ran the same model comparison method to test for differences in average trait expression and trait variation between invasive New Zealand populations (Auckland [year of introduction = 1960], Hamilton [1978], Whangarei [2002], and Edgecumbe [2007]) and their respective Australian source region (i.e., Tenterfield; Supplementary Tables 12–14). All models contained population as a fixed-effect, while individual lizard ID was included as a random intercept. The model which allowed both among- and within-individual variance to differ among populations (i.e., Model 4) provided the best fit to the exploratory behavior and activity data (Supplementary Tables 12–13), whereas the model which only allowed within-individual variance to differ among regions (i.e., Model 3) provided a slightly better fit to the boldness data (Supplementary Table 14). Nevertheless, as the difference between the fit of two models was small and, for the sake of consistency, we extracted variance estimates for each population for each behavioral trait from the model which allowed both among- and within-individual variance to differ among populations (i.e., Model 4) to statistically compare how behavioral variance changed during the New Zealand invasion. We report V_A_ and V_W_ for each population, mean-trait level differences between populations, as well as the effect size of the difference in repeatability and variance components for each pairwise population contrast (∆R, ∆ V_A_ and ∆V_W_; Supplementary Tables 15–21).

### Reporting summary

Further information on research design is available in the Nature Research Reporting Summary linked to this article.

## Supplementary information

Supplementary Information

Reporting Summary

## Data Availability

All data used in this study have been submitted to the Bridges data repository^78^ (10.26180/18851036.v1). Source data are provided with this paper.

## Source data

Source Data

## Code availability

All code used in this study have been submitted to the Bridges data repository^78^ (10.26180/18851036.v1).
