# Supplementary material for: Biological invasions as a selective filter driving behavioral divergence
Source: Nat Commun. 2022 Oct 11;13:5996. doi: 10.1038/s41467-022-33755-2 (PMC9553908; doi:10.1038/s41467-022-33755-2)
Supplement: Supplementary file 1 — Supplementary Information [file 41467_2022_33755_MOESM1_ESM.pdf]

# Supplementary Information for

## Biological invasions as a selective filter driving behavioral divergence

David G. Chapple[1,#]*, Annalise C. Naimo[1]*, Jack A. Brand[1]*, Marcus Michelangeli[1,2,3], Jake M. Martin[1,3], Celine T. Goulet[1], Dianne H. Brunton[4], Andrew Sih[2], and Bob B.M. Wong[1].

[#]Correspondence to: David.Chapple@monash.edu

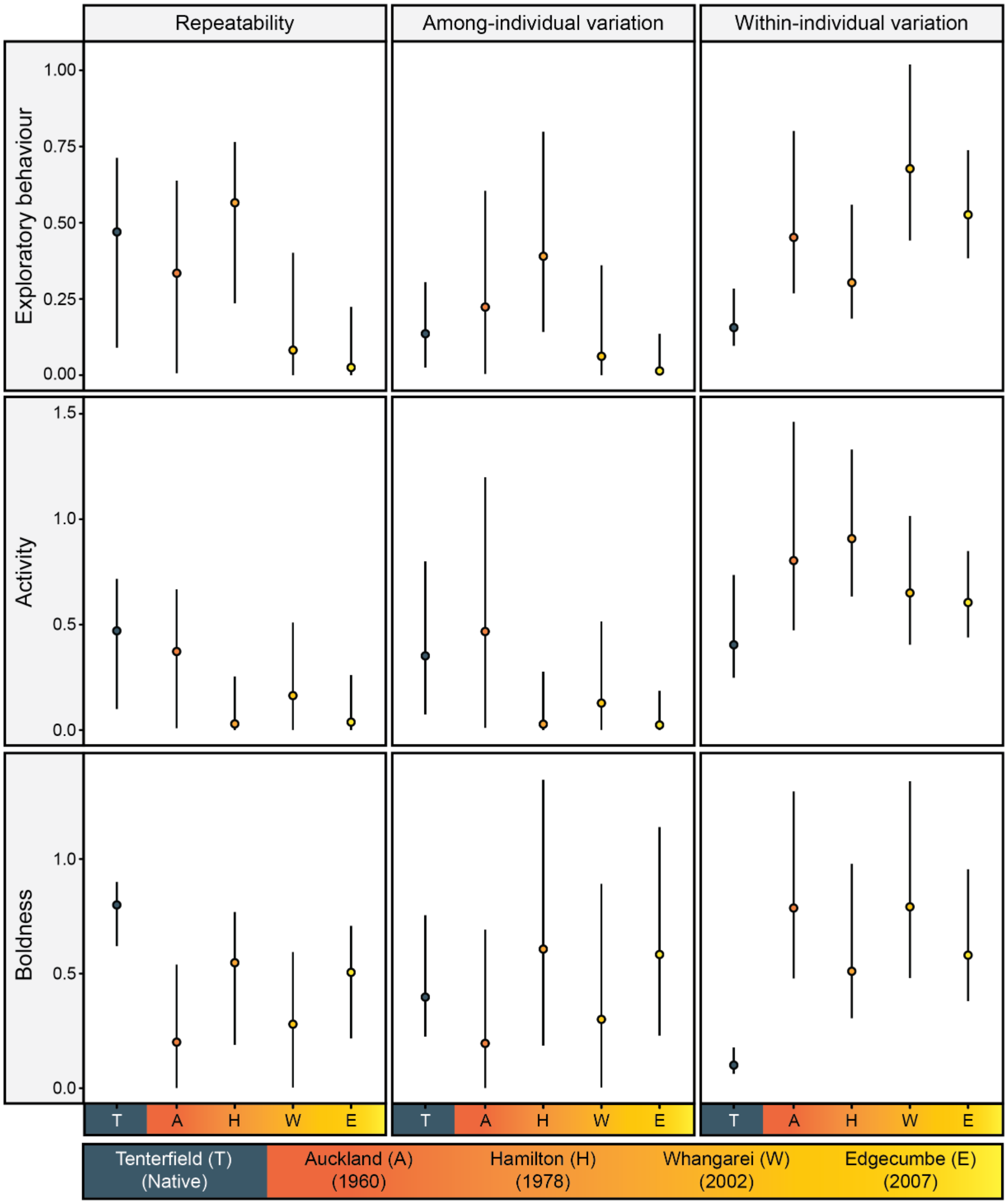


**Supplementary Fig. 1.** Adjusted repeatability and variance estimates (among- and within-individual) for exploratory behavior (i.e. time spent exploring the barrier), activity (i.e. the number of transitions between grid squares), and boldness (i.e. re-emergence latency; axis inverted) of skinks from the native Tenterfield source population (grey; $n$ = 30 skinks), and invasive New Zealand skinks from Auckland (red; $n$ = 31), Hamilton (orange; $n$ = 43), Whangarei (light-orange; $n$ = 33), and Edgecumbe (yellow; $n$ = 36). For each graph, filled circles represent the median variance/repeatability estimates extracted from linear mixed-effects models, while vertical error bars denote 95% credible intervals. Source data are provided as a Source Data file.

**Supplementary Table 1.** The number of lizards collected from each population across the species native (i.e., Australia) and invasive (i.e., Hawaii, Lord Howe Island, and New Zealand) ranges.

| Region | Population | Establishment date | Number of skinks in each population | Number of skinks in each region |
|---|---|---|---|---|
| Australia | Brisbane | Native | 27 | 167 |
| Australia | Coffs Harbour | Native | 81 | |
| Australia | Sydney | Native | 29 | |
| Australia | Tenterfield | Native | 30 | |
| Hawaii | Koke'e | ~1960 | 39 | 118 |
| Hawaii | Honolulu | 1905 | 37 | |
| Hawaii | Volcano | ~1960 | 42 | |
| Lord Howe Island | Boardwalk | ~1980 | 26 | 92 |
| Lord Howe Island | Middle Beach | ~1980 | 30 | |
| Lord Howe Island | North Bay | ~1980 | 36 | |
| New Zealand | Auckland | ~1960 | 31 | 143 |
| New Zealand | Hamilton | 1978 | 43 | |
| New Zealand | Whangarei | 2002 | 33 | |
| New Zealand | Edgecumbe | 2007 | 36 | |

**Supplementary Table 2.** Experimental timeline of behavioral trials. Skinks underwent two trials of activity, exploratory behavior, and boldness, each four days apart. Note: some lizards performed behavioral tests seven days apart due to logistical constraints.

| | Experimental day | | | | | | |
|---|---|---|---|---|---|---|---|
| **Week** | **Day 1** | **Day 2** | **Day 3** | **Day 4** | **Day 5** | **Day 6** | **Day 7** |
| 1 | Activity trial 1 | | | | Activity trial 2 | | |
| 2 | | Exploration trial 1 | | | | Exploration trial 2 | |
| 3 | | | Boldness trial 1 | | | | Boldness trial 1 |

**Supplementary Table 3.** Model comparison table comparing the relative fits of four models investigating differences in exploration among both native and invasive regions.

| Model | WAIC | Se WAIC | Δ WAIC | LOO | Se LOO | Δ LOO |
|---|---|---|---|---|---|---|
| Both variance | 2403.0 | 63.1 | 0 | 2432.8 | 64.9 | 0 |
| Within model | 2403.9 | 60.3 | –0.9 | 2433.1 | 62.0 | –0.3 |
| Null model | 2430.8 | 59.7 | –27.8 | 2451.3 | 60.7 | –18.5 |
| Among model | 2442.4 | 61.3 | –39.4 | 2461.0 | 62.1 | –28.2 |

**Supplementary Table 4**. Model comparison table comparing the relative fits of four models investigating differences in activity rates among both native and invasive regions.

| Model | WAIC | Se WAIC | Δ WAIC | LOO | Se LOO | Δ LOO |
|---|---|---|---|---|---|---|
| Both variance | 2505.6 | 47.7 | 0 | 2550.2 | 50.1 | 0 |
| Within model | 2518.2 | 46.3 | –12.6 | 2561.3 | 48.3 | –11.1 |
| Among model | 2532.8 | 48.4 | –27.2 | 2569.5 | 50.0 | –19.3 |
| Null model | 2538.8 | 47.1 | –33.2 | 2578.8 | 49.0 | –28.6 |

**Supplementary Table 5.** Model comparison table comparing the relative fits of four models investigating differences in boldness among both native and invasive regions.

| Model | WAIC | Se WAIC | Δ WAIC | LOO | Se LOO | Δ LOO |
|---|---|---|---|---|---|---|
| Both variance | 2177.4 | 43.0 | 0 | 2232.1 | 45.1 | 0 |
| Within model | 2183.0 | 42.9 | –5.6 | 2234.4 | 44.9 | –2.3 |
| Null model | 2272.1 | 42.2 | –94.7 | 2311.0 | 43.7 | –78.9 |
| Among model | 2277.9 | 42.9 | –100.5 | 2313.7 | 44.4 | –81.6 |

**Supplementary Table 6**. Model summary of average differences in exploratory behavior between Australia (i.e. intercept; *n* = 167 skinks), and Hawaii (*n* = 118) Lord Howe Island (*n* = 92), and New Zealand (*n* = 143).

| Term | Estimate | SE | Lower 95% CI | Upper 95% CI |
|---|---|---|---|---|
| Intercept | **–0.644** | **0.165** | **–0.955** | **–0.293** |
| Hawaii | **1.099** | **0.248** | **0.573** | **1.563** |
| Lord Howe Island | **0.606** | **0.252** | **0.076** | **1.077** |
| New Zealand | **0.954** | **0.233** | **0.465** | **1.398** |

Estimates in bold are those with 95% CI's that did not include zero.

**Supplementary Table 7**. Model summary of average differences in activity rates between Australia (i.e. intercept; *n* = 167 skinks), and Hawaii (*n* = 118) Lord Howe Island (*n* = 92), and New Zealand (*n* = 143).

| Term | Estimate | SE | Lower 95% CI | Upper 95% CI |
|---|---|---|---|---|
| Intercept | –0.173 | 0.165 | –0.501 | 0.165 |
| Hawaii | 0.364 | 0.258 | –0.152 | 0.868 |
| Lord Howe Island | –0.205 | 0.258 | –0.724 | 0.314 |
| New Zealand | 0.434 | 0.233 | –0.029 | 0.895 |

**Supplementary Table 8.** Model summary of average differences in boldness between Australia (i.e. intercept; *n* = 167 skinks), and Hawaii (*n* = 118) Lord Howe Island (*n* = 92), and New Zealand (*n* = 143). Note: as boldness was measured as re-emergence latencies, lower scores indicate bolder lizards.

| Term | Estimate | SE | Lower 95% CI | Upper 95% CI |
|---|---|---|---|---|
| Intercept | 0.128 | 0.085 | –0.044 | 0.299 |
| Hawaii | 0.030 | 0.129 | –0.216 | 0.292 |
| Lord Howe Island | 0.269 | 0.150 | –0.029 | 0.566 |
| New Zealand | **–0.597** | **0.137** | **–0.864** | **–0.325** |

Estimates in bold are those with 95% CI's that did not include zero.

**Supplementary Table 9.** Adjusted repeatability (R) and variance estimates (i.e., among-individual [$V_A$] and within-individual [$V_W$]) for exploratory behavior in Australia (*n* = 167 skinks), and Hawaii (*n* = 118) Lord Howe Island (*n* = 92), and New Zealand (*n* = 143).

| Region | Estimate | Value | Lower 95% CI | Upper 95% CI |
|---|---|---|---|---|
| Australia | R | 0.44 | 0.31 | 0.56 |
| Australia | $V_A$ | 0.34 | 0.22 | 0.47 |
| Australia | $V_W$ | 0.34 | 0.27 | 0.42 |
| Hawaii | R | 0.15 | 0.00 | 0.29 |
| Hawaii | $V_A$ | 0.13 | 0.00 | 0.27 |
| Hawaii | $V_W$ | 0.64 | 0.48 | 0.82 |
| Lord Howe Island | R | 0.09 | 0.00 | 0.24 |
| Lord Howe Island | $V_A$ | 0.09 | 0.00 | 0.24 |
| Lord Howe Island | $V_W$ | 0.79 | 0.58 | 1.01 |
| New Zealand | R | 0.18 | 0.00 | 0.31 |
| New Zealand | $V_A$ | 0.15 | 0.00 | 0.26 |
| New Zealand | $V_W$ | 0.57 | 0.43 | 0.72 |

**Supplementary Table 10.** Adjusted repeatability (R) and variance estimates (i.e., among-individual [$V_A$] and within-individual [$V_W$]) for activity rates in Australia (*n* = 167 skinks), and Hawaii (*n* = 118) Lord Howe Island (*n* = 92), and New Zealand (*n* = 143).

| Region | Estimate | Value | Lower 95% CI | Upper 95% CI |
|---|---|---|---|---|
| Australia | R | 0.44 | 0.32 | 0.57 |
| Australia | $V_A$ | 0.38 | 0.26 | 0.52 |
| Australia | $V_W$ | 0.37 | 0.29 | 0.45 |
| Hawaii | R | 0.44 | 0.28 | 0.58 |
| Hawaii | $V_A$ | 0.57 | 0.33 | 0.84 |
| Hawaii | $V_W$ | 0.62 | 0.46 | 0.80 |
| Lord Howe Island | R | 0.31 | 0.12 | 0.50 |
| Lord Howe Island | $V_A$ | 0.32 | 0.10 | 0.54 |
| Lord Howe Island | $V_W$ | 0.60 | 0.43 | 0.81 |
| New Zealand | R | 0.09 | 0.00 | 0.22 |
| New Zealand | $V_A$ | 0.08 | 0.00 | 0.20 |
| New Zealand | $V_W$ | 0.73 | 0.57 | 0.90 |

**Supplementary Table 11.** Adjusted repeatability (R) and variance estimates (i.e., among-individual [$V_A$] and within-individual [$V_W$]) for boldness in Australia (*n* = 167 skinks), and Hawaii (*n* = 118) Lord Howe Island (*n* = 92), and New Zealand (*n* = 143).

| Region | Estimate | Value | Lower 95% CI | Upper 95% CI |
|---|---|---|---|---|
| Australia | R | 0.58 | 0.46 | 0.70 |
| Australia | $V_A$ | 0.38 | 0.26 | 0.51 |
| Australia | $V_W$ | 0.25 | 0.19 | 0.31 |
| Hawaii | R | 0.29 | 0.10 | 0.47 |
| Hawaii | $V_A$ | 0.23 | 0.07 | 0.40 |
| Hawaii | $V_W$ | 0.53 | 0.39 | 0.69 |
| Lord Howe Island | R | 0.10 | 0.00 | 0.30 |
| Lord Howe Island | $V_A$ | 0.12 | 0.00 | 0.35 |
| Lord Howe Island | $V_W$ | 1.00 | 0.68 | 1.33 |
| New Zealand | R | 0.42 | 0.27 | 0.56 |
| New Zealand | $V_A$ | 0.57 | 0.32 | 0.83 |
| New Zealand | $V_W$ | 0.75 | 0.58 | 0.95 |

**Supplementary Table 12.** Model comparison table comparing the relative fits of four models investigating differences in exploratory behavior between native Tenterfield population, and individual invasive New Zealand populations (Auckland, Hamilton, Whangarei, and Edgecumbe).

| Model | WAIC | Se WAIC | Δ WAIC | LOO | Se LOO | Δ LOO |
|---|---|---|---|---|---|---|
| Both variance | 696.4 | 39.8 | 0 | 713.0 | 41.5 | 0 |
| Within model | 706.6 | 39.0 | –10.2 | 719.7 | 40.7 | –6.7 |
| Among model | 724.3 | 42.7 | –27.9 | 729.6 | 42.6 | –16.6 |
| Null model | 728.9 | 41.1 | –32.5 | 734.2 | 41.6 | –21.2 |

**Supplementary Table 13.** Model comparison table comparing the relative fits of four models investigating differences in activity rates between native Tenterfield population, and individual invasive New Zealand populations (Auckland, Hamilton, Whangarei, and Edgecumbe).

| Model | WAIC | Se WAIC | Δ WAIC | LOO | Se LOO | Δ LOO |
|---|---|---|---|---|---|---|
| Both variance | 873.4 | 25.8 | 0 | 882.5 | 26.4 | 0 |
| Among model | 878.1 | 25.3 | –4.7 | 883.5 | 25.7 | –1 |
| Within model | 889.4 | 26.1 | –16.0 | 895.1 | 26.6 | –12.6 |
| Null model | 891.5 | 26.3 | –18.1 | 895.2 | 26.7 | –12.7 |

**Supplementary Table 14.** Model comparison table comparing the relative fits of four models investigating differences in boldness between native Tenterfield population, and individual invasive New Zealand populations (Auckland, Hamilton, Whangarei, and Edgecumbe).

| Model | WAIC | Se WAIC | Δ WAIC | LOO | Se LOO | Δ LOO |
|---|---|---|---|---|---|---|
| Within model | 742.9 | 21.7 | 0 | 764.8 | 22.5 | 0 |
| Both variance | 749.4 | 21.6 | –6.5 | 769.6 | 22.2 | –4.8 |
| Null model | 791.0 | 20.3 | –48.1 | 809.1 | 21.4 | –44.3 |
| Among model | 799.3 | 19.9 | –56.4 | 814.9 | 20.8 | –50.1 |

**Supplementary Table 15**. Model summary of average differences in exploratory behavior between the native Tenterfield population (i.e., intercept; *n* = 30 skinks), and individual invasive New Zealand populations (Auckland [established 1960; *n* = 31], Hamilton [established 1978; *n* = 43], Whangarei [established 2002; *n* = 33], and Edgecumbe [established 2007; *n* = 36]).

| Term | Estimate | SE | Lower 95% CI | Upper 95% CI |
|---|---|---|---|---|
| Intercept | **–1.284** | **0.085** | **–1.450** | **–1.111** |
| Auckland | **1.625** | **0.154** | **1.321** | **1.930** |
| Hamilton | **1.266** | **0.161** | **0.945** | **1.584** |
| Whangarei | **1.700** | **0.141** | **1.413** | **1.974** |
| Edgecumbe | **1.638** | **0.120** | **1.402** | **1.873** |

Estimates in bold are those with 95% CI’s that did not include zero.

**Supplementary Table 16**. Model summary of average differences in activity between the native Tenterfield population (i.e., intercept; *n* = 30 skinks), and individual invasive New Zealand populations (Auckland [established 1960; *n* = 31], Hamilton [established 1978; *n* = 43], Whangarei [established 2002; *n* = 33], and Edgecumbe [established 2007; *n* = 36]).

| Term | Estimate | SE | Lower 95% CI | Upper 95% CI |
|---|---|---|---|---|
| Intercept | **–0.632** | **0.134** | **–0.895** | **–0.362** |
| Auckland | **0.471** | **0.217** | **0.048** | **0.899** |
| Hamilton | **0.542** | **0.183** | **0.179** | **0.899** |
| Whangarei | **0.816** | **0.176** | **0.466** | **1.161** |
| Edgecumbe | **1.133** | **0.161** | **0.814** | **1.445** |

Estimates in bold are those with 95% CI's that did not include zero.

**Supplementary Table 17**. Model summary of average differences in boldness between the native Tenterfield population (i.e., intercept; *n* = 30 skinks), and individual invasive New Zealand populations (Auckland [established 1960; *n* = 31], Hamilton [established 1978; *n* = 43], Whangarei [established 2002; *n* = 33], and Edgecumbe [established 2007; *n* = 36]). Note: as boldness was measured as re-emergence latencies, lower scores indicate bolder lizards.

| Term | Estimate | SE | Lower 95% CI | Upper 95% CI |
|---|---|---|---|---|
| Intercept | 0.319 | 0.121 | 0.074 | 0.550 |
| Auckland | –0.180 | 0.187 | –0.544 | 0.188 |
| Hamilton | **–0.617** | **0.214** | **–1.036** | **–0.189** |
| Whangarei | **–0.389** | **0.194** | **–0.758** | **–0.003** |
| Edgecumbe | **–0.389** | **0.191** | **–0.753** | **–0.009** |

Estimates in bold are those with 95% CI's that did not include zero.

**Supplementary Table 18.** Adjusted repeatability (R) and variance estimates (i.e., among-individual [$V_A$] and within-individual [$V_W$]) for exploratory behavior within each population in the invasive New Zealand lineage (i.e., Tenterfield [native; *n* = 30 skinks], Auckland [established 1960; *n* = 31], Hamilton [established 1978; *n* = 43], Whangarei [established 2002; *n* = 33], and Edgecumbe [established 2007; *n* = 36]).

| Region | Estimate | Value | Lower 95% CI | Upper 95% CI |
|---|---|---|---|---|
| Tenterfield | R | 0.45 | 0.15 | 0.75 |
| Tenterfield | $V_A$ | 0.14 | 0.00 | 0.27 |
| Tenterfield | $V_W$ | 0.16 | 0.09 | 0.26 |
| Auckland | R | 0.33 | 0.00 | 0.60 |
| Auckland | $V_A$ | 0.24 | 0.00 | 0.52 |
| Auckland | $V_W$ | 0.47 | 0.24 | 0.75 |
| Hamilton | R | 0.55 | 0.27 | 0.78 |
| Hamilton | $V_A$ | 0.41 | 0.12 | 0.77 |
| Hamilton | $V_W$ | 0.32 | 0.17 | 0.52 |
| Whangarei | R | 0.12 | 0.00 | 0.35 |
| Whangarei | $V_A$ | 0.10 | 0.00 | 0.30 |
| Whangarei | $V_W$ | 0.69 | 0.43 | 0.99 |
| Edgecumbe | R | 0.05 | 0.00 | 0.18 |
| Edgecumbe | $V_A$ | 0.03 | 0.00 | 0.11 |
| Edgecumbe | $V_W$ | 0.54 | 0.37 | 0.72 |

**Supplementary Table 19.** Adjusted repeatability (R) and variance estimates (i.e., among-individual [$V_A$] and within-individual [$V_W$]) for activity within each population in the invasive New Zealand lineage (i.e., Tenterfield [native; *n* = 30 skinks], Auckland [established 1960; *n* = 31], Hamilton [established 1978; *n* = 43], Whangarei [established 2002; *n* = 33], and Edgecumbe [established 2007; *n* = 36]).

| Region | Estimate | Value | Lower 95% CI | Upper 95% CI |
|---|---|---|---|---|
| Tenterfield | R | 0.46 | 0.15 | 0.75 |
| Tenterfield | $V_A$ | 0.38 | 0.00 | 0.71 |
| Tenterfield | $V_W$ | 0.43 | 0.23 | 0.69 |
| Auckland | R | 0.36 | 0.00 | 0.63 |
| Auckland | $V_A$ | 0.50 | 0.00 | 1.05 |
| Auckland | $V_W$ | 0.85 | 0.42 | 1.36 |
| Hamilton | R | 0.06 | 0.00 | 0.20 |
| Hamilton | $V_A$ | 0.06 | 0.00 | 0.22 |
| Hamilton | $V_W$ | 0.93 | 0.59 | 1.27 |
| Whangarei | R | 0.19 | 0.00 | 0.46 |
| Whangarei | $V_A$ | 0.16 | 0.00 | 0.43 |
| Whangarei | $V_W$ | 0.66 | 0.38 | 0.97 |
| Edgecumbe | R | 0.06 | 0.00 | 0.22 |
| Edgecumbe | $V_A$ | 0.04 | 0.00 | 0.15 |
| Edgecumbe | $V_W$ | 0.62 | 0.43 | 0.83 |

**Supplementary Table 20.** Adjusted repeatability (R) and variance estimates (i.e., among-individual [$V_A$] and within-individual [$V_W$]) for boldness within each population in the invasive New Zealand lineage (i.e., Tenterfield [native; *n* = 30 skinks], Auckland [established 1960; *n* = 31], Hamilton [established 1978; *n* = 43], Whangarei [established 2002; *n* = 33], and Edgecumbe [established 2007; *n* = 36]).

| Region | Estimate | Value | Lower 95% CI | Upper 95% CI |
|---|---|---|---|---|
| Tenterfield | R | 0.79 | 0.65 | 0.91 |
| Tenterfield | $V_A$ | 0.42 | 0.20 | 0.69 |
| Tenterfield | $V_W$ | 0.10 | 0.06 | 0.16 |
| Auckland | R | 0.21 | 0.00 | 0.49 |
| Auckland | $V_A$ | 0.23 | 0.00 | 0.59 |
| Auckland | $V_W$ | 0.81 | 0.45 | 1.24 |
| Hamilton | R | 0.53 | 0.24 | 0.80 |
| Hamilton | $V_A$ | 0.65 | 0.15 | 1.26 |
| Hamilton | $V_W$ | 0.54 | 0.27 | 0.89 |
| Whangarei | R | 0.28 | 0.00 | 0.55 |
| Whangarei | $V_A$ | 0.33 | 0.00 | 0.78 |
| Whangarei | $V_W$ | 0.82 | 0.45 | 1.27 |
| Edgecumbe | R | 0.49 | 0.25 | 0.73 |
| Edgecumbe | $V_A$ | 0.61 | 0.20 | 1.10 |
| Edgecumbe | $V_W$ | 0.60 | 0.35 | 0.90 |

**Supplementary Table 21**. The effect size (± 95 % CI) of the magnitude difference in among-individual variation ($\Delta V_A$), within-individual variation ($\Delta V_W$), and repeatability ($\Delta R$) of exploration, activity, and boldness for each population in the invasive New Zealand lineage (i.e., Tenterfield [AUS; *n* = 30 skinks], Auckland [AUK; *n* = 31], Hamilton [HAM; *n* = 43], Whangarei [WGI; *n* = 33], and Edgecumbe [EDG; *n* = 36]).

| | Exploration | | | Activity | | | Boldness | | |
|---|---|---|---|---|---|---|---|---|---|
| Contrast | $\Delta V_A$ | $\Delta V_W$ | $\Delta R$ | $\Delta V_A$ | $\Delta V_W$ | $\Delta R$ | $\Delta V_A$ | $\Delta V_W$ | $\Delta R$ |
| AUS – AUK | -0.10 | **-0.31** | 0.13 | -0.12 | -0.42 | 0.10 | 0.19 | **-0.71** | **0.57** |
| | (-0.44, 0.21) | **(-0.61, -0.04)** | (-0.31, 0.59) | (-0.86, 0.54) | (-1.04, 0.09) | (-0.34, 0.58) | (-0.31, 0.62) | **(-1.14, -0.33)** | **(0.25, 0.88)** |
| AUS – HAM | -0.27 | -0.16 | -0.10 | 0.32 | **-0.50** | **0.40** | -0.23 | **-0.44** | 0.26 |
| | (-0.63, 0.09) | (-0.38, 0.04) | (-0.50, 0.31) | (-0.07, 0.72) | **(-0.94, -0.06)** | **(0.04, 0.70)** | (-0.89, 0.39) | **(-0.79, -0.15)** | (-0.05, 0.60) |
| AUS – WGI | 0.05 | **-0.53** | 0.34 | 0.22 | -0.24 | 0.27 | 0.09 | **-0.72** | **0.51** |
| | (-0.21, 0.27) | **(-0.84, -0.23)** | (-0.06, 0.67) | (-0.26, 0.69) | (-0.63, 0.17) | (-0.16, 0.65) | (-0.46, 0.61) | **(-1.16, -0.32)** | **(0.18, 0.84)** |
| AUS – EDG | 0.12 | **-0.37** | **0.40** | 0.33 | -0.19 | **0.39** | -0.19 | **-0.50** | **0.30** |
| | (-0.04, 0.28) | **(-0.57, -0.17)** | **(0.05, 0.69)** | (-0.02, 0.72) | (-0.51, 0.14) | **(0.03, 0.70)** | (-0.74, 0.33) | **(-0.80, -0.24)** | **(0.02, 0.59)** |
| AUK – HAM | -0.17 | 0.15 | -0.22 | 0.44 | -0.08 | 0.30 | -0.42 | 0.27 | -0.32 |
| | (-0.63, 0.28) | (-0.17, 0.51) | (-0.67, 0.18) | (-0.09, 1.09) | (-0.69, 0.55) | (-0.04, 0.66) | (-1.15, 0.23) | (-0.27, 0.82) | (-0.71, 0.10) |
| AUK – WGI | 0.15 | -0.22 | 0.21 | 0.34 | 0.18 | 0.17 | -0.10 | -0.01 | -0.06 |
| | (-0.22, 0.53) | (-0.62, 0.18) | (-0.19, 0.60) | (-0.30, 1.03) | (-0.36, 0.83) | (-0.29, 0.60) | (-0.73, 0.50) | (-0.62, 0.61) | (-0.49, 0.39) |
| AUK – EDG | 0.21 | -0.06 | 0.28 | 0.45 | 0.23 | 0.29 | -0.38 | 0.21 | -0.28 |
| | (-0.06, 0.54) | (-0.39, 0.27) | (-0.06, 0.62) | (-0.06, 1.07) | (-0.25, 0.82) | (-0.07, 0.63) | (-0.98, 0.22) | (-0.27, 0.75) | (-0.66, 0.09) |

| | | | | | | | | | |
|---|---|---|---|---|---|---|---|---|---|
| HAM – WGI | 0.32 | **-0.37** | **0.43** | -0.10 | 0.26 | -0.13 | 0.32 | -0.28 | 0.25 |
| | (-0.06, 0.73) | **(-0.74, -0.04)** | **(0.08, 0.75)** | (-0.47, 0.20) | (-0.20, 0.74) | (-0.47, 0.16) | (-0.44, 1.05) | (-0.87, 0.25) | (-0.16, 0.68) |
| HAM – EDG | **0.38** | -0.21 | **0.50** | 0.01 | 0.31 | -0.01 | 0.04 | -0.06 | 0.04 |
| | **(0.08, 0.75)** | (-0.47, 0.06) | **(0.19, 0.77)** | (-0.16, 0.23) | (-0.08, 0.74) | (-0.24, 0.19) | (-0.67, 0.80) | (-0.52, 0.41) | (-0.35, 0.41) |
| WGI – EDG | 0.07 | 0.15 | 0.07 | 0.12 | 0.05 | 0.12 | -0.28 | 0.22 | -0.22 |
| | (-0.11, 0.31) | (-0.20, 0.49) | (-0.16, 0.36) | (-0.13, 0.45) | (-0.34, 0.41) | (-0.16, 0.47) | (-0.91, 0.41) | (-0.30, 0.77) | (-0.60, 0.19) |

Contrasts in bold are those with 95% CI's that did not include zero.
