# Supplementary material for: Biological invasions as a selective filter driving behavioral divergence
Source: Nat Commun. 2022 Oct 11;13:5996. doi: 10.1038/s41467-022-33755-2 (PMC9553908; doi:10.1038/s41467-022-33755-2)
Supplement: Supplementary file 2 — Reporting Summary [file 41467_2022_33755_MOESM2_ESM.pdf]

nature portfolio

Corresponding author(s): David G. Chapple
Last updated by author(s): Sep 28, 2022

Reporting Summary

Nature Portfolio wishes to improve the reproducibility of the work that we publish. This form provides structure for consistency and transparency in reporting. For further information on Nature Portfolio policies, see our Editorial Policies and the Editorial Policy Checklist.

Statistics

For all statistical analyses, confirm that the following items are present in the figure legend, table legend, main text, or Methods section.

n/a	Confirmed	
☐	☒	The exact sample size (*n*) for each experimental group/condition, given as a discrete number and unit of measurement
☐	☒	A statement on whether measurements were taken from distinct samples or whether the same sample was measured repeatedly
☐	☒	The statistical test(s) used AND whether they are one- or two-sided<br>*Only common tests should be described solely by name; describe more complex techniques in the Methods section.*
☐	☒	A description of all covariates tested
☐	☒	A description of any assumptions or corrections, such as tests of normality and adjustment for multiple comparisons
☐	☒	A full description of the statistical parameters including central tendency (e.g. means) or other basic estimates (e.g. regression coefficient) AND variation (e.g. standard deviation) or associated estimates of uncertainty (e.g. confidence intervals)
☐	☒	For null hypothesis testing, the test statistic (e.g. *F*, *t*, *r*) with confidence intervals, effect sizes, degrees of freedom and *P* value noted<br>*Give P values as exact values whenever suitable.*
☐	☒	For Bayesian analysis, information on the choice of priors and Markov chain Monte Carlo settings
☒	☐	For hierarchical and complex designs, identification of the appropriate level for tests and full reporting of outcomes
☒	☐	Estimates of effect sizes (e.g. Cohen's *d*, Pearson's *r*), indicating how they were calculated

Our web collection on statistics for biologists contains articles on many of the points above.

Software and code

Policy information about availability of computer code

Data collection	The key-logging software BORISv8 was used to score the videos of the behavioural trials. Friard, O. & Gamba, M. BORIS: a free, versatile open-source event-logging software for video/audio coding and live observations. Methods in Ecology and Evolution 7, 1325–1330 (2016).
Data analysis	The data analysis was conducted in R version 3.6.2. R is available from https://www.r-project.org/.

For manuscripts utilizing custom algorithms or software that are central to the research but not yet described in published literature, software must be made available to editors and reviewers. We strongly encourage code deposition in a community repository (e.g. GitHub). See the Nature Portfolio guidelines for submitting code & software for further information.

Data

Policy information about availability of data

All manuscripts must include a data availability statement. This statement should provide the following information, where applicable:

- Accession codes, unique identifiers, or web links for publicly available datasets
- A description of any restrictions on data availability
- For clinical datasets or third party data, please ensure that the statement adheres to our policy

All data and code used in this study have been submitted to the Bridges data repository (https://doi.org/10.26180/18851036.v2)

Human research participants

Policy information about studies involving human research participants and Sex and Gender in Research.

Reporting on sex and gender	NA
Population characteristics	NA
Recruitment	NA
Ethics oversight	NA

Note that full information on the approval of the study protocol must also be provided in the manuscript.

Field-specific reporting

Please select the one below that is the best fit for your research. If you are not sure, read the appropriate sections before making your selection.

☐ Life sciences ☐ Behavioural & social sciences ☒ Ecological, evolutionary & environmental sciences

For a reference copy of the document with all sections, see nature.com/documents/nr-reporting-summary-flat.pdf

Ecological, evolutionary & environmental sciences study design

All studies must disclose on these points even when the disclosure is negative.

Study description	We compared the behaviour of the delicate skink (Lampropholis delicata) between its native Australian range, and its invasive range (Lord Howe Island, New Zealand, Hawaiian Islands).
Research sample	Adult male delicate skinks (Lampropholis delicata) collected from eastern Australia, Lord Howe Island, New Zealand, and the Hawaiian Islands.
Sampling strategy	We collected 27-81 lizards per population, and 92-167 lizards from each region. Previous studies have demonstrated that this sample size is sufficient to account for behavioural variation within populations.
Data collection	All skinks were tested for activity, exploration, and boldness following previously established methods for Lampropholis skinks. All lizards were tested in a controlled temperature room maintained at 22.5 ± 1 °C. Skinks first performed: (i) an activity assay testing non-directed activity, (ii) a novel obstacle test testing exploratory behavior, and (iii) an anti-predator assay testing boldness following a simulated predator attack. All assays were performed in fixed order to reduce carryover effects. Each assay was repeated after four days to measure both among- and within-individual behavioral variation. Trials were video-recorded (JVC Everio GZ-E100) from above and scored blind to experimental conditions using the key-logging software BORIS. All equipment was thoroughly washed between each trial with scentless detergent. Further, as Lampropholis skinks are known to modify their behaviors following large meals, we ensured that lizards were not fed for 24 h prior to each behavioral assay. Data collection was completed by ACN.
Timing and spatial scale	Fieldwork was conducted in Australia (November 2015; December 2018), Lord Howe Island (November-December 2018), New Zealand (December 2017), and the Hawaiian Islands (June-July 2019).
Data exclusions	No data were excluded from the analyses.
Reproducibility	All behavioural trials were conducted twice for each lizard, as quantifying behavioural repeatability was a core focus of the study.
Randomization	Lizards were assigned to the population from which they were collected.
Blinding	Trials were video-recorded (JVC Everio GZ-E100) from above and scored blind to experimental conditions.

Did the study involve field work? ☒ Yes ☐ No

Field work, collection and transport

Field conditions	Fieldwork was conducted during conditions where the delicate skinks were active and basking.
Location	The fieldwork was conducted in Australia (Brisbane [27°27S, 152°58E], Coffs Harbour [30°21S, 153°05E], Sydney [33°54S, 151°11E], Tenterfield [28°51S, 152°03E]), Lord Howe Island (31°30S, 159°03E), Hawaii (Honolulu, Oahu

nature portfolio | reporting summary

March 2021

	[21°18N, 157°49W]; Volcano, Hawaii [19°26N, 155°13W]; Koke'e, Kauai [22°07N, 159°39W]), and New Zealand (Auckland [36°43S, 174°41E], Hamilton [37°47S, 175°15E], Whangarei [35°49S, 174°30E], Edgecumbe [37°58S, 176°49E]).
Access & import/export	Most collection locations were in urban locations, with easy access. The research was conducted with the appropriate collection permits (Hawaii: K2019-4044cc and EX-19-18, Lord Howe Island: LHIB 09/18, Australia: SL102160 [New South Wales] and 10008946 [Victoria]).
Disturbance	Lizards were caught using methods that targeted active, basking lizards. This minimized the disturbance to the field work locations.

Reporting for specific materials, systems and methods

We require information from authors about some types of materials, experimental systems and methods used in many studies. Here, indicate whether each material, system or method listed is relevant to your study. If you are not sure if a list item applies to your research, read the appropriate section before selecting a response.

Materials & experimental systems

n/a	Involved in the study	
☒	☐	Antibodies
☒	☐	Eukaryotic cell lines
☒	☐	Palaeontology and archaeology
☐	☒	Animals and other organisms
☒	☐	Clinical data
☒	☐	Dual use research of concern

Methods

n/a	Involved in the study	
☒	☐	ChIP-seq
☒	☐	Flow cytometry
☒	☐	MRI-based neuroimaging

Animals and other research organisms

Policy information about studies involving animals; ARRIVE guidelines recommended for reporting animal research, and Sex and Gender in Research

Laboratory animals	N/A
Wild animals	Adult, male delicate skinks (Lampropholis delicata) with complete tails (SVL>tail length) were used to avoid the well-documented effects of tail loss and gravidity on skink behaviour. Skinks were caught using hand capture, mealworm fishing. Animals were transported by plane (using a commercial pet transport company JetPets) to Monash University, Melbourne, Australia (Australian and Lord Howe Island populations) or the Center for Aquatic Biology and Aquaculture, University of California, Davis (Hawaiian populations). For the New Zealand populations, the lizards were transported by car to Massey University, Auckland. At the conclusion of the experiments, delicate skinks from Australia and Lord Howe Island were retained in the laboratory and used in other research projects. As the species is invasive in New Zealand and Hawaii, at the conclusion of the experiments, lizards collected from these locations were humanely killed following a protocol approved by the relevant animal ethics committees (intraperitoneal injection of sodium pentabarbitone).
Reporting on sex	Only adult male delicate skinks were used. Previous studies have shown that gravidity influences behaviour, and female skinks were gravid during the study period.
Field-collected samples	The research was conducted with the appropriate collection permits (Hawaii: K2019-4044cc and EX-19-18, Lord Howe Island: LHIB 09/18, Australia: SL102160 [New South Wales] and 10008946 [Victoria]). Skinks were housed in groups of up to seven individuals in plastic containers (300 × 370 mm) within temperature-controlled rooms (13:11 h light/dark cycle, maintained at 22.5 ± 1 °C). Each container was fitted with small plastic pots and newspapers for shelter. A basking area was created by placing heat-tape under a terracotta tile at one end of each housing container. This created a thermal gradient (22–32 °C) that allowed thermoregulation from 0800 to 1700 h. Similarly, UV lighting was provided above containers from 0800–1800 h. Skinks were fed a diet of crickets (Acheta domesticus) dusted in a vitamin supplement (Reptivite), three times a week, and water was made available ad libitum. Skinks were acclimated to laboratory conditions for at least 1 week before experiments.
Ethics oversight	The research was conducted with the appropriate animal ethics approvals (University of California Animal Ethics Committee, Davis Protocol No. 211194, Monash University School of Biological Sciences Animal Ethics Committee approval No. 16736, Massey University Animal Ethics Committee approval No. MUAEC17/76).

Note that full information on the approval of the study protocol must also be provided in the manuscript.
